# Effects of a New Flavonoid and Myo-Inositol Supplement on Some Biomarkers of Cardiovascular Risk in Postmenopausal Women: A Randomized Trial

**DOI:** 10.1155/2014/653561

**Published:** 2014-08-31

**Authors:** Rosario D'Anna, Angelo Santamaria, Maria Letizia Cannata, Maria Lieta Interdonato, Grazia Maria Giorgianni, Roberta Granese, Francesco Corrado, Alessandra Bitto

**Affiliations:** ^1^Department of Pediatric, Gynecological, Microbiological, and Biomedical Sciences, University of Messina, 98125 Messina, Italy; ^2^Messina University Hospital, A.O.U. “G. Martino”, 98125 Messina, Italy; ^3^Department of Clinical and Experimental Medicine, University of Messina, 98125 Messina, Italy

## Abstract

*Background and Aim*. Cardiovascular risk is increased in women with menopause and metabolic syndrome. Aim of this study was to test the effect of a new supplement formula, combining cocoa polyphenols, myo-inositol, and soy isoflavones, on some biomarkers of cardiovascular risk in postmenopausal women with metabolic syndrome.* Methods and Results*. A total of 60 women were enrolled and randomly assigned (*n* = 30 per group) to receive the supplement (NRT: 30 mg of cocoa polyphenols, 80 mg of soy isoflavones, and 2 gr of myo-inositol), or placebo for 6 months. The study protocol included three visits (baseline, 6, and 12 months) for the evaluation of glucose, triglycerides, and HDL-cholesterol (HDL-C), adiponectin, visfatin, resistin, and bone-specific alkaline phosphatase (bone-ALP). At 6 months, a significant difference between NRT and placebo was found for glucose (96 ± 7 versus 108 ± 10 mg/dL), triglycerides (145 ± 14 versus 165 ± 18 mg/dL), visfatin (2.8 ± 0.8 versus 3.7 ± 1.1 ng/mL), resistin (27 ± 7 versus 32 ± 8 *µ*g/L), and b-ALP (19 ± 7 versus 15 ± 5 *µ*g/mL). No difference in HDL-C concentrations nor in adiponectin levels between groups was reported at 6 months.* Conclusions.* The supplement used in this study improves most of the biomarkers linked to metabolic syndrome. This Trial is registered with NCT01400724.

## 1. Introduction

Cardiovascular disease (CVD) events remain the leading cause of mortality and are a major cause of morbidity and disability in both genders worldwide [1]. CVDs cover a collection of various heart and vascular diseases, among these hypertension, coronary heart, and cerebrovascular diseases represent major public health problems. It is well known that in postmenopausal CVD risk is increased, since the protective effects exerted by estrogens are missing. It is a matter of fact that following menopause, negative changes in blood pressure, glucose level, and lipid profile are frequent, and characterize the so called “metabolic syndrome”, responsible for increased CVD risk [2]. Furthermore, the majority of postmenopausal women experience an undesirable weight gain. CVDs have multiple causes; however, most of the events originate from the complications of atherosclerosis, a pathophysiological process that can be prevented by dietary habit [3]. Evidence from epidemiological studies indicates a positive association between reduction in the incidence of CVD and consumption of plant-based foods such as fruit and vegetables [4]. In addition, the relationship between the amount of polyphenol-rich food consumption (fruit and fruit juices, tea, wine, and cocoa) and chronic diseases, supports a protective effect of these compounds upon CVD [5]. The meta-analysis by Desch and colleagues [6] has confirmed the blood pressure-lowering capacity of flavonol-rich cocoa products, in a large set of trials. It has been estimated that a 3 mmHg reduction in systolic blood pressure (SBP) would reduce the relative risk of stroke mortality by 8%, coronary artery disease (CAD) mortality by 5%, and all-cause of mortality by 4% [7]. Other effects of cocoa-derived products include the improved platelet function [8] and the reduction of LDL-cholesterol [4], the major atherogenic lipoprotein.

Besides cocoa flavanols, many other dietary substances have a proven efficacy in reducing CVD risk markers; in previous studies we already shown the protective effects of two naturally occurring molecules, the isoflavone genistein [9] and myo-inositol [10, 11], produced by the human body from glucose. Inositol is a polyol which may be considered a second messenger of insulin [12], and myo-inositol is one of its nine isomers, capable of reducing insulin resistance, blood pressure, and improving lipid profile in a small cohort of postmenopausal women affected by metabolic syndrome [10, 11]. The soy-derived isoflavone genistein acting as a natural selective estrogen receptor modulator (SERM) has a proven efficacy on markers of CVD risk [9] and in reducing bone loss in postmenopausal women [13]. Very recently, genistein has shown to reduce insulin resistance, blood pressure, and homocysteine and it improved lipid profile in a cohort of women with metabolic syndrome [14].

In light of these previous experiences and observations, the aim of this study was to test a new supplement formula, combining cocoa polyphenols, myo-inositol, and soy isoflavones, in postmenopausal women with metabolic syndrome. The rationale for this combination therapy is to offer a natural replacement therapy (NRT) that might improve the metabolic conditions in postmenopausal women. In particular, cocoa polyphenols are antioxidants; inositol is an insulin sensitizing agent and soy isoflavones have a positive effect also on bone metabolism.

## 2. Subjects and Methods

### 2.1. Enrollment and Randomization to Treatments

A 12-month, randomized, open-label study was performed. The study protocol was consistent with the principles of the Declaration of Helsinki and has been approved by the Ethics Committee of the University of Messina, Italy. All participants gave written informed consent. Women (*n* = 60) were recruited at the Menopause Outpatients of the University of Messina. Following the criteria for inclusion, women had to be 50 to 60 years old, postmenopausal for at least 12 months at baseline, and diagnosed with metabolic syndrome. Based on the NCEP ATP III [15], the diagnosis of metabolic syndrome in women requires three or more of the five following criteria: (1) waist circumference ≥ 88 cm; (2) triglycerides ≥ 150 mg/dL or on drug treatment for elevated triglycerides; (3) HDL-C < 50 mg/dL or on drug treatment for reduced HDL-C; (4) fasting glucose ≥ 100 mg/dL or on drug treatment for hyperglycemia; (5) blood pressure ≥ 130/85 mmHg or on antihypertensive drug. At enrollment a complete family history was obtained and physical examination and routine laboratory evaluation were performed. Almost all the women were hypertensive (55/60) and in treatment with antihypertensive agents.

Exclusion criteria were clinical or laboratory evidence of confounding systemic diseases (e.g., chronic renal or hepatic failure and chronic inflammatory diseases); breast affections or familiar history of breast disease; CVD defined as documented myocardial infarction, ischemic heart disease, coronary heart bypass, coronary angioplasty, cerebral thromboembolism, peripheral amputations, and coagulopathy; use of oral or transdermal estrogen, progestin, androgens, selective estrogen receptor modulators, or other steroids; treatment in the preceding six months with polyunsaturated n-3 fatty acids supplements and nonsteroidal anti-inflammatory drugs (NSAIDs); smoking habit of more than 2 cigarettes daily. Furthermore, subjects were advised to report on occasional use of NSAIDs to the study investigators. Additional exclusion criteria included history of alcohol or drug abuse, being enrolled in another clinical study, proven hypersensitivity to the study drugs, and concomitant major diseases. A computer generated randomization with sequence random permuted blocks was used to minimize differences between groups, due to the limited number of women. Subjects were assigned to the intervention group (NRT: *n* = 30) or to the placebo group (*n* = 30).

All participants were counseled on a Mediterranean-style diet composed of 25% to 30% energy from fat, less than 10% from saturated fatty acids, 55% to 60% from carbohydrates, and 15% from protein, with a cholesterol intake less than 300 mg/d and fiber intake of 35 g/d or greater. We used this diet to avoid interference with the lipid profile. Diet adherence was evaluated in all participants during each follow-up visit. Soy products or other dietary supplements were prohibited whereas legumes' intake was suggested to be kept constant overtime. At least 150 minutes per week of moderately intense physical activity (walking or cycling) was recommended.

The treatment (herein after NRT) was given for 6 months and it was composed of 30 mg of cocoa polyphenols, 80 mg of soy isoflavones, and 2 grams of myo-inositol. Both NRT and placebo were in powder. In the following 6-month period only diet was recommended to both groups. Women were followed monthly with telephone calls to reinforce the adherence to the intervention.

The study protocol included three visits (baseline, 6, and 12 months) for evaluation of serum glucose, triglycerides, and HDL-C together with some adipokines: adiponectin, visfatin, and resistin; and a marker of bone turnover: bone-ALP.

### 2.2. Determination of the Study Variables

Primary outcome was the improvement (as a 20% change from baseline) of at least one of the parameters that characterize metabolic syndrome: blood pressure, waist circumference, HDL-cholesterol, triglycerides, and fasting glucose. Secondary outcome was the improvement of the studied markers as adiponectin, resistin, visfatin, and bone-ALP.

Fasting glucose was measured using routine colorimetric method (normal range 65–110 mg/dL). HDL-C and triglycerides were measured by using a routine enzymatic method (SGM Italia, Italy).

Adiponectin, resistin (all from DRG International Inc., Marburg, Germany), bone-specific alkaline phosphatase (IDS, ltd, Fountain Hills, AZ), and visfatin (Ucsn Life Science, Inc., Houston, TX) were measured using enzyme-linked immunosorbent assay kits from serum samples. Adiponectin limit of detection was 0.2 *μ*g/mL with an intra-assay CV of 7.5% and an interassay CV of 6.5%. Resistin limit of detection was 0.012 ng/mL with an intra-assay CV of 5.2% and an interassay CV of 7%. Visfatin limit of detection was 6.3 pg/mL with an intra-assay CV of 9% and an interassay CV of 10%. Bone-ALP limit of detection was 0.7 *μ*g/L with an intra-assay CV of 4.5% and an interassay CV of 5.8%.

### 2.3. Adverse Events

Participants were also followed and monitored by their general practitioners (who served as external monitors), who received a detailed synopsis of the trial and were unaware of the treatment arm. At clinical visit every 6 months, women were asked about symptoms. Standard clinical evaluations and routinary laboratory analyses were done every 6 months. All unfavorable and unintended clinical effects were considered adverse events and were evaluated for severity, duration, seriousness, and relation to the study drug and outcome.

### 2.4. Statistical Analysis

The analysis-of-variance (ANOVA) for repeated measures was performed to analyze treatment effects on groups and time and the unpaired *t*-test was used to compare mean differences in continuous variables intra- and intergroups. A *P* value of 0.05 or less was considered statistically significant. A post hoc power calculation analysis demonstrated over 95% power when considering as primary endpoint either glycemia or triglycerides, with an alpha error of 0.05. Statistical analysis was performed by using Statistical Package for Social Science (SPSS Statistics 17.0 Chicago, IL) software.

## 3. Results

At the beginning of the study, the 2 groups were comparable for age, BMI, months from menopause, waist circumferences, and blood pressure (Table 1); and for clinical characteristics: serum glucose, HDL-cholesterol, and triglycerides (Table 2).

**Figure 1:**
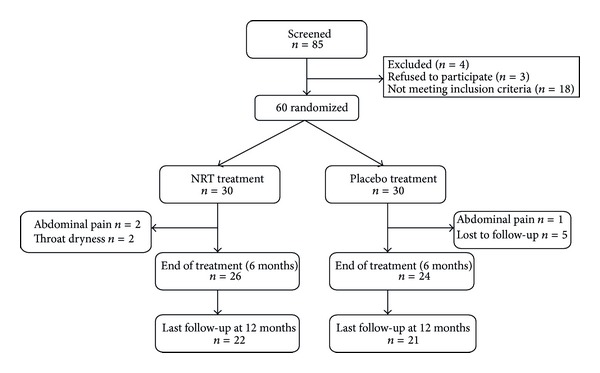
Study flow chart.

At the end of the 6 months treatment period, only 26 in the NRT group and 24 in the placebo group remained in the study (Figure 1). In the following six-month period other dropouts occurred, thus only 22 in the treated group and 21 in the placebo group completed the study. Reasons for withdrawal were abdominal pain (3 cases) and throat dryness (2 cases) and other reasons were not reported.

**Figure 2:**
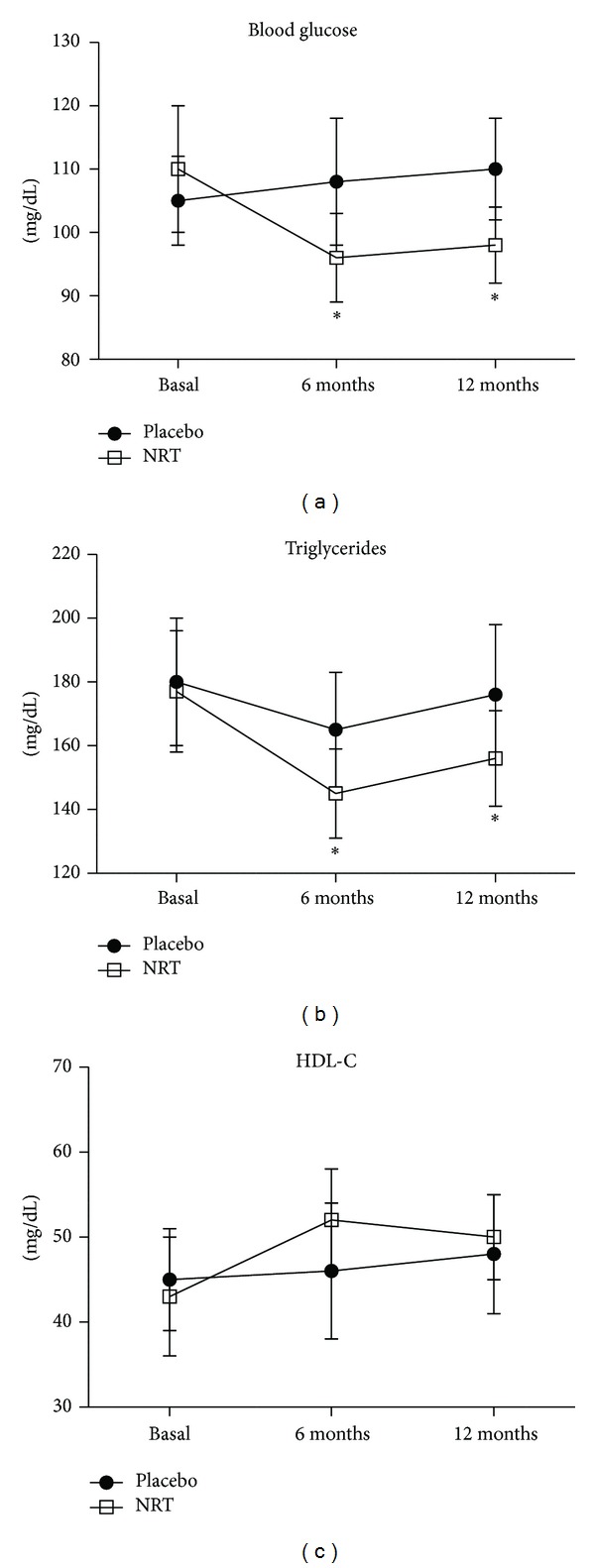
Glucose, triglycerides and HDL-cholesterol blood levels through the study. *P < 0.05 versus basal.

**Figure 3:**
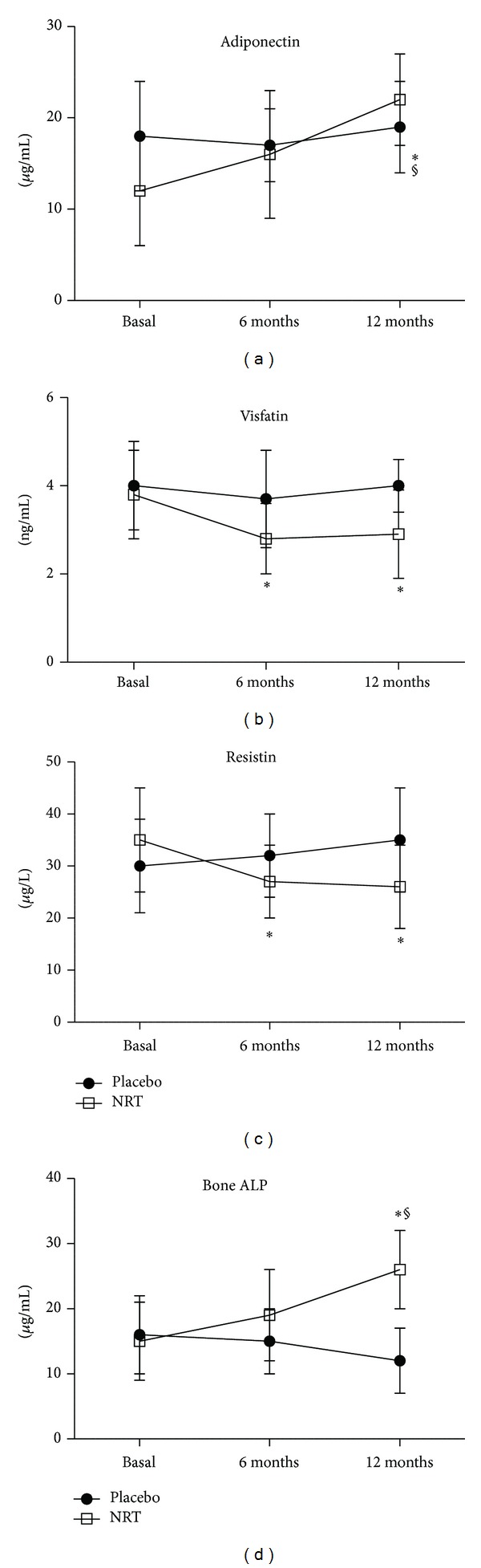
Adiponectin, visfatin, resistin, and bone-ALP blood levels through the study. *P < 0.05 versus basal; ^§^ *P* < 0.05 versus placebo.

At 6 months, a significant difference between NRT and placebo was found for glucose, triglycerides, b-ALP, visfatin, and resistin as reported in Table 2 and Figures 2 and 3. No difference in HDL-C concentrations nor in adiponectin levels between groups was reported at 6 months (Table 2 and Figures 2 and 3). At 12 months, after 6 months from the end of the treatment period, in addition to glucose, triglycerides, b-ALP, visfatin, and resistin, also adiponectin levels were significantly changed in NRT group, compared to placebo (Table 2 and Figures 2 and 3).

The intra-group analysis revealed that only HDL-C and adiponectin were not affected by NRT treatment at the end of the 6 months, while all the other studied variables were significantly changed from basal values (Table 2 and Figures 2 and 3). In the placebo group after 6 months a significant difference from basal values was observed for triglycerides and adiponectin, probably due to diet, despite no changes in BMI were noted in the enrolled subjects (Table 2 and Figures 2 and 3).

## 4. Discussion

The results of this study demonstrate a protective effect on cardiovascular markers following 6 months administration of a combination of myo-inositol, soy isoflavones, and cocoa polyphenols, in postmenopausal women with metabolic syndrome.

A limit of this study is the number of women enrolled, which reduced the statistical significance of the outcomes; in spite of all that, some interesting results have been obtained. One of these was the persistence, in the six months follow-up period, of the positive results achieved during the six-month treatment period; this data concerns all the markers studied. No differences were highlighted in BMI and waist circumference between and within groups, but this may be due to the short treatment period and somewhat to the low adherence to diet. In the treated group, serum glucose and triglycerides values significantly decreased after six months, maintaining in the subsequent 6 months a significant difference from basal levels. These results are in accordance with other studies, in which each component of the supplement of this trial was used [10, 14, 16]. No difference in HDL-C was shown either between groups or from 6-month to basal values; this data is confirmed by some studies in which only cocoa polyphenols were used [5, 17], but not by others [10, 14, 16], in which a significant difference compared to control group was shown, when each single component of the supplement studied was used. Blood pressure didn't significantly change through the study period, although significant differences were highlighted in other studies in which either myo-inositol or cocoa polyphenols were used [6, 10]; probably the limited number of women studied has negatively influenced this outcome. More interesting were the data about biomarkers which usually depend on insulin resistance. In particular, serum adiponectin levels were significantly increased, after 12 months, in the treated group compared to the control group and also increased at 12 months from basal values in the same group. This result is in accordance with a recent study of our group where genistein aglycone was used [14]. Adiponectin is an adipocyte-specific, secreted protein that sensitizes the liver and muscle to the action of insulin [18]. It is the only adipocyte-derived hormone to be downregulated in the insulin-resistant state, so the levels of adiponectin strongly correlate with basal insulin levels and insulin sensitivity [19]. This explains why low concentrations of adiponectin are associated with increased prevalence of metabolic syndrome, especially in postmenopausal women [20]. If an improvement of insulin resistance is expeceted using isoflavones, because they act through estrogen receptors; or myo-inositol, because of its insulin-like effect [10, 11]; an insulin sensitizing effect is not yet clear for cocoa polyphenols. According to Grassi and coworkers [21], a possible explanation might be found in the positive effect that cocoa extracts have on endothelium-dependent relaxation; in fact, since insulin sensitivity may be in part considered dependent on NO availability, cocoa polyphenols may improve NO production and insulin sensitivity as a consequence [22]. For resistin and visfatin a significant difference occurred only in the treated group, in which both markers were significantly reduced after 6 months of supplement assumption, reaching a plateau until the 12th month. Resistin, is a peptide hormone produced by adipocytes that is more highly expressed in omental and abdominal subcutaneous white fat [23]. In postmenopausal, and in obese women, resistin levels nearly double, representing the hormone that links obesity to diabetes [24]; in addition, a close association of resistin to metabolic syndrome has been recently established [25]. Visfatin role is controversial, but recent evidences have shown increased serum levels in overweight/obese, type-2 diabetics, metabolic syndrome, and CVD patients [26]. Kim and coworkers [27] have suggested that visfatin may act as the underlying pathophysiological trigger for metabolic syndrome in postmenopausal women and as a marker of abdominal fat deposition and tissue inflammation. The here reported significant reduction in visfatin values, is in agreement with our recent findings in postmenopausal women with metabolic syndrome taking the isoflavone genistein for 12 months [14]. Among the biomarkers evaluated, bone-ALP was also included. This molecule is synthesized by the osteoblasts and it is considered a specific marker of bone formation [28]. In this study, the group receiving the combination formula showed increased levels of bone-ALP over time, with significant differences compared to the placebo group after 12 months and a significant difference with respect to basal values. This result is in accordance with a previous study [29], showing that 54 mg of genistein improved bone turnover, prevented osteoporosis, and increased bone mass density in postmenopausal osteopenic women. Thus, we may hypothesize that probably isoflavones have determined an improvement in bone turnover; however, an experimental study [30] has shown that also myo-inositol is essential for osteogenesis and bone formation.

In conclusion, the supplement used in this study has shown to improve most of the biomarkers linked to metabolic syndrome, suggesting a possible reduction of CVD risk. Furthermore, this study has shown that the positive effects of the supplement may last in the subsequent six months, together with an increase in bone formation. Further studies are warranted to reproduce the present data in a larger cohort of postmenopausal women with metabolic syndrome.

## Figures and Tables

**Table 1:** Demographic and clinical characteristics of the study groups (mean ± SD).

	NRT *n* = 30 (basal)	Placebo *n* = 30 (basal)	*P*	NRT *n* = 26 (6 months)	Placebo *n* = 24 (6 months)	*P*
Age (years)	56.3 ± 3.8	55.5 ± 4.8	0.3			
Menopause (months)	95.1 ± 61.6	75.6 ± 57	0.2			
BMI (Kg/m²)	31.9 ± 3.8	33.6 ± 3.9	0.1	30.7 ± 3.9	33.3 ± 3.3	0.2
Systolic blood pressure (mmHg)	123.6 ± 12	132.6 ± 20	0.09	124 ± 9.1	121.6 ± 9.8	0.3
Diastolic blood pressure (mmHg)	80 ± 8	81.5 ± 11.9	0.3	73.6 ± 11.2	70 ± 6.3	0.17
Waist circumference (cm)	98.6 ± 6.2	100.8 ± 12.6	0.2	98.8 ± 6	96.3 ± 6.9	0.17

**Table 2:** Biomarkers evaluation through the study (mean ± SD).

		Glucose (mg/dL)	HDL-C (mg/dL)	Triglycerides (mg/dL)	B-ALP (*µ*g/mL)	Adiponectin (*µ*g/mL)	Visfatin (ng/mL)	Resistin (*µ*g/L)
NRT	Basal	110 ± 10	44 ± 7	177 ± 19	15 ± 6	18 ± 6	3.8 ± 1	35 ± 10
	6 months	96 ± 7*	50 ± 6	145 ± 14*	19 ± 7**	17 ± 4	2.8 ± 0.8***	27 ± 7**
	12 months	98 ± 6^##^	50 ± 5	156 ± 15^§^	26 ± 6^##^	19 ± 5^##^	2.9 ± 1^##^	26 ± 8^§§^
Basal versus 6 months	*P* value	<0.001	0.06	<0.001	<0.001	0.48	0.002	0.0016

Placebo	Basal	105 ± 7	45 ± 6	180 ± 20	16 ± 6	22 ± 5	4 ± 1	30 ± 9
	6 months	108 ± 10	46 ± 8	165 ± 18	15 ± 5	16 ± 7	3.7 ± 1.1	32 ± 8
	12 months	110 ± 8	48 ± 7	176 ± 22	12 ± 5	12 ± 6	4 ± 0.6	35 ± 10
Basal versus 6 months	*P* value	0.23	0.62	0.008	0.53	0.0013	0.32	0.42

*P < 0.001 versus placebo 6 months; *P* = 0.004 versus placebo 6 and 12 months; **P = 0.02 versus placebo 6 months; ***P = 0.001 versus placebo 6 months; ^##^ *P* < 0.001 versus placebo 12 months; ^§^ *P* = 0.004 versus placebo 12 months; ^§§^ *P* = 0.009 versus placebo 12 months.
